# Veterinarians’ Perspectives on the Antimicrobial Resistance (AMR) Dashboard: A Survey of Needs and Preferences to Inform Development

**DOI:** 10.3390/vetsci12100940

**Published:** 2025-09-28

**Authors:** Abraham Joseph Pellissery, Thomas Denagamage, Maura Pedersen, Subhashinie Kariyawasam

**Affiliations:** 1 Comparative, Diagnostic, and Population Medicine, College of Veterinary Medicine, University of Florida, Gainesville, FL 32608, USA; a.pellissery@ufl.edu (A.J.P.);; 2 Bronson Animal Disease Diagnostic Laboratory, Florida Department of Agriculture and Consumer Services, Kissimmee, FL 34741, USA; 3 Large Animal Clinical Sciences, College of Veterinary Medicine, University of Florida, Gainesville, FL 32608, USA; tdenagamage@ufl.edu

**Keywords:** antimicrobial resistance, AMR dashboard, One Health, veterinarians

## Abstract

Antimicrobial resistance (AMR) poses a significant global threat to human and animal health, necessitating robust surveillance and stewardship tools. While existing systems address aspects of veterinary AMR, a comprehensive, user-centric dashboard for U.S. veterinarians remains a critical unmet need. This study aimed to identify U.S. veterinarians’ preferences and perceived needs for such a dashboard, to help guide its design and development. A cross-sectional survey was conducted between January and March 2024, targeting U.S. veterinarians through professional channels. The survey instrument captured demographics, experiences with existing tools, preferences for data types and visualizations, desired technical specifications, and open-ended feedback. Of the 677 respondents, a near-unanimous consensus (over 75%) emerged on the importance of functionalities like antimicrobial stewardship education, off-label use guidance, surveillance data, and empirical treatment support. Over 70% expressed comfort sharing aggregated geographic and de-identified animal data. A strong preference was observed for making the dashboard accessible by veterinary colleges (78.87%), diagnostic laboratories (72.61%), and federal agencies (USDA: 71.47%, CDC: 66.67%, FDA: 62.11%), indicating a desire for a collaborative, authoritative system. The findings provide a robust foundation for developing a U.S. veterinary AMR dashboard. Future phases should adopt an iterative, user-centered design, incorporating qualitative research with diverse stakeholders and piloting a prototype with preferred institutional partners. This approach will ensure a trusted, sustainable tool that effectively translates surveillance data into actionable insights for improved animal and public health.

## Simple Summary

Antimicrobial resistance (AMR) is a growing threat to animal and human health. Between January and March 2024, U.S. veterinarians were surveyed to understand their needs and preferences for a new online tool, called an AMR dashboard, which can help track and manage resistance. We found that veterinarians strongly desire a dashboard that offers educational content, guidance on antibiotic use, and data for surveillance and treatment decisions. They prefer that this dashboard be managed collaboratively by veterinary colleges, diagnostic labs, and federal agencies, such as the USDA, CDC, and FDA, with data shared in a way that protects privacy. This indicates a clear demand for a reliable, centralized system. Future steps involve (a) conducting further research with diverse animal health stakeholders and (b) piloting a prototype dashboard with key partners, to ensure the system is practical, trustworthy, and widely adopted, ultimately improving animal and public health.

## 1. Introduction

Antimicrobial resistance (AMR) is a growing global challenge that impacts human, animal, and environmental health, highlighting the importance of the One Health approach in addressing this issue [1,2,3,4,5,6]. Significant attention has been focused on AMR in human healthcare [7,8]. However, the role of veterinary medicine in the emergence and spread of resistant pathogens is equally important, but often overlooked, in existing AMR surveillance systems [9,10]. Responsible antimicrobial use in veterinary practice is vital for ensuring animal welfare and preventing the rise in resistant pathogens, making effective tracking and reporting in this field essential for understanding the full impact of AMR [11,12]. Currently, global AMR tracking in veterinary medicine is inconsistent, with notable gaps in data collection, reporting, and collaboration across borders [6,12]. In many areas, surveillance efforts are either underdeveloped or lack standardization, complicating the comparison of trends among different countries and hindering effective interventions. Moreover, the absence of a unified reporting framework on a global scale makes it challenging to accurately monitor resistance patterns and antimicrobial usage in animal populations. Despite these barriers, initiatives like the World Health Organization’s (WHO) One Health approach are striving to enhance veterinary AMR surveillance by promoting integrated data sharing across human, animal, and environmental health sectors [1,2,3].

Enhancing the tracking and reporting processes in veterinary medicine are essential for the fight against AMR globally and for shaping evidence-based policies and antimicrobial stewardship [13]. AMR dashboards serve as vital centralized tools that help collect, analyze, and visualize AMR trends, enabling data-driven decision-making in stewardship and surveillance [14]. By incorporating laboratory results, field observations, and treatment records, these dashboards better equip veterinarians, researchers, and policymakers to monitor resistance patterns, detect emerging threats, and devise targeted interventions that uphold antimicrobial efficacy and improve clinical outcomes. In this regard, a Veterinary AMR Dashboard plays a crucial role in consolidating data on resistance trends within the veterinary sector. To bolster this effort, the World Organization for Animal Health (WOAH, formerly known as OIE) has launched the ANIMUSE (Animal Anti-Microbial USE) system, a global platform aimed at collecting and visualizing veterinary antimicrobial usage (AMU) data [15]. This platform enables countries to upload, review, and track AMU trends over time, featuring built-in data validation and adaptable reporting options. Multiple initiatives across the globe are working to improve the surveillance of antimicrobial resistance (AMR) in animals. While some, like the EFSA AMR Explorer and the UK’s APHA, focus on livestock and the food chain, other platforms, such as Europe’s EARS-Vet and China’s CARPet, are specifically dedicated to monitoring AMR in companion animals and wildlife [16,17,18,19,20,21,22,23].

In the United States, several agencies, including the United States Department of Agriculture (USDA), Centers for Disease Control and Prevention (CDC), Food and Drug Administration (FDA), and National Institutes of Health (NIH), have implemented various programs to monitor and report AMR in animals. A key initiative in this area is the National Antimicrobial Resistance Monitoring System (NARMS), which has been operational since 1996. This program, jointly managed by the FDA, CDC, and USDA, emphasizes a One Health approach toward addressing AMR [24]. NARMS tracks changes in the antimicrobial susceptibility of enteric bacteria in ill individuals, retail meats, and food animals throughout the country. This program is essential for protecting public health, as it provides valuable information on emerging bacterial resistance, distinguishes between resistant and susceptible infections, and evaluates the effectiveness of strategies aimed at preventing the spread of resistance. The FDA uses data from NARMS to guide regulatory decisions that ensure antibiotics remain effective for both humans and animals. In addition, the National Database of Antibiotic Resistant Organisms (NDARO), hosted by the National Center for Biotechnology Information, centralizes AMR data to support real-time surveillance and research on pathogenic organisms [25,26]. The Veterinary Laboratory Investigation and Response Network (Vet-LIRN) partners with veterinary diagnostic labs to share scientific data, strengthen lab capacity, and enhance both human and animal health outcomes [27]. The National Animal Health Laboratory Network (NAHLN), led by the USDA’s Animal and Plant Health Inspection Service (APHIS), regularly monitors AMR in animal pathogens through U.S. veterinary diagnostic laboratories [27,28]. Meanwhile, the Companion Animal Veterinary Surveillance Network (CAVSNET) collects and analyzes data from companion animal clinics to track antimicrobial use and resistance by species, syndrome, and drug type, enabling benchmarking and supporting responsible antimicrobial stewardship in veterinary practice [29].

While NARMS has played a significant role in One Health surveillance of AMR in the U.S. at the national level, there are still notable gaps and limitations to address [12]:

Exclusion of companion animals: It does not currently gather AMR data from pets like dogs and cats, despite rising antimicrobial use and the risk of zoonotic transmission.

Limited pathogen focus: The program primarily concentrates on a few enteric pathogens, including *Salmonella* spp., *Campylobacter* spp., *Escherichia coli* (mostly commensal), and *Enterococcus* spp. This excludes many important veterinary pathogens associated with diseased animals.

Testing data limitations: NARMS mainly relies on phenotypic testing data (minimum inhibitory concentrations and breakpoints). Although whole genome sequencing (WGS) data has started to be included, particularly for human and meat isolates, the development of animal-sector pathogen genomics remains limited, hindering our understanding of resistance gene transmission dynamics.

Sampling bias: The current retail meat sampling process is confined to specific cuts (like chicken breasts, ground turkey, and pork chops) and select retailers, which may not provide a comprehensive overview of resistance throughout the food chain. Additionally, USDA FSIS sampling in slaughterhouses may not accurately reflect on-farm AMR ecology, thus affecting early resistance detection.

Delayed data publication: Although NARMS provides interactive dashboards for monitoring AMR in *Salmonella* and antibiotic use in animals, the data often has a publication lag of 1–2 years, diminishing its effectiveness for timely decision-making.

Lack of AMU and AMR integration: While both AMR and antimicrobial usage (AMU) are monitored in U.S. agriculture, they are not directly linked at the species or pathogen level. This disconnect limits the ability to analyze correlations between antibiotic usage and emerging resistance trends.

Insufficient One Health integration: Although NARMS contributes to the U.S. One Health AMR response, it does not encompass environmental surveillance (like water, waste, and soil), nor does it provide cross-sector dashboards or integrated analytics that visually link trends across human, animal, and food sectors.

To address these challenges, the USDA’s Animal and Plant Health Inspection Service (APHIS) launched a funding initiative from 2022 to 2024 focused on the development and enhancement of AMR dashboards [30]. This program aims to monitor AMR trends in domesticated animals, such as livestock, poultry, and pets, providing tens of millions in joint funding to academic institutions, public organizations, and other collaborators. While AMR dashboards serve as essential tools for tracking resistance trends, optimizing antimicrobial use, and guiding policy decisions, they encounter significant hurdles related to data confidentiality and operational issues. A primary concern is maintaining the confidentiality of sensitive information, especially in veterinary medicine and agriculture, where antimicrobial usage (AMU) data is often linked to specific farms, producers, or veterinarians. Striking a balance between safeguarding this confidential data and allowing meaningful analysis and cross-sector collaboration is challenging. Additional obstacles include ensuring data quality, completeness, and standardization across different regions, as well as inconsistencies in reporting among various stakeholders, from healthcare providers to farmers. Without standardized data and secure protocols, AMR dashboards may fall short in effectively identifying resistance patterns or informing interventions. To make AMR dashboards reliable and actionable tools in the fight against AMR, it is vital to tackle these data confidentiality issues, while also enhancing data infrastructure and fostering collaboration across sectors. As part of the USDA APHIS initiative to create and utilize AMR dashboard tools for better access to information on AMR in domesticated animals, we conducted a survey of U.S. veterinarians. Our goal was to capture their perspectives and preferences regarding AMR dashboards for veterinary use, which will inform the AMR Dashboard development efforts of The National Institute of Antimicrobial Resistance Research and Education (NIAMRRE).

## 2. Materials and Methods

### 2.1. Study Design and Setting

Between 1 January 2024 and 31 March 2024, we conducted a comprehensive cross-sectional survey aimed at evaluating the needs and preferences of veterinarians concerning AMR dashboards (Supplemental File S1: Survey Instrument). The targeted animal species/categories included bovine, swine poultry, companion animals (canine, feline), equine, small ruminants (ovine, caprine, cervine, etc.), wildlife, aquatic (aquaculture), and bees (apiculture). The survey targeted U.S. veterinarians and was disseminated through various channels, including veterinary teaching hospitals, specialty organization listservs, and professional veterinary organizations. Additionally, it was shared through veterinary diagnostic laboratory client distribution lists and relevant websites to enhance reach and participation. The survey was hosted on Qualtrics XM (Qualtrics, Provo, UT, USA), ensuring a user-friendly interface for participants. Responses were collected anonymously, with participation implying consent, thereby safeguarding the rights and privacy of all involved.

### 2.2. Survey Instrument

In collaboration with USDA APHIS, we developed a structured questionnaire tailored to capture a wide range of insights. The questionnaire comprised five distinct sections: (1) demographics of the respondents, (2) experiences with existing AMR dashboards, (3) preferences for data types and visualizations, (4) desired technical specifications and usability features, and (5) an avenue for open-ended feedback. The question formats included a mixture of multiple-choice questions, Likert scale items to gauge levels of agreement or satisfaction, rankings of tasks to prioritize preferences, and opportunities for free-text responses. Before launching the survey, we pilot-tested the instrument with five AMR experts to enhance clarity and ensure face validity.

### 2.3. Data Collection and Distribution

The survey was distributed using a multi-faceted approach to maximize participation and gather a diverse set of responses. Veterinary Teaching hospitals, professional networks, specialty organizations’ listservs, and veterinary diagnostic laboratory client portals were utilized to reach potential respondents effectively.

#### Sample Size Calculation

Stratified random sampling (proportional) was performed (Table 1), with an expected response rate of 20% using the U.S. veterinarian number statistics from the American Veterinary Medical Association [31].

### 2.4. Participants and Inclusion Criteria

To participate in the survey, individuals needed to be veterinary professionals practicing in the U.S. with experience in administering antibiotics for animal treatment. There were no geographical or sectoral restrictions beyond the requirement that participants be U.S. veterinarians, nor were there limitations based on the participants’ level of experience. This inclusivity aimed to gather a comprehensive understanding of the veterinary landscape related to AMR.

### 2.5. Data Analysis

Quantitative responses were analyzed using descriptive statistics, including frequencies and proportions. For qualitative data derived from open-ended responses, thematic content analysis was conducted to identify and categorize recurring themes concerning user needs, challenges, and suggestions regarding AMR dashboards.

### 2.6. Ethical Considerations

Participation in the survey was entirely voluntary, and critical measures were implemented to ensure confidentiality of respondents. No personal identifiers were collected to maintain anonymity, and all response data were stored securely with access restricted to authorized personnel only. This research project was classified as “exempt research” (Protocol #ET00019861) by the University of Florida Institutional Review Board, allowing us to proceed with the study while adhering to ethical standards and guidelines for human subject research. The data collected will be utilized solely for research and AMR dashboard development purposes, contributing to the broader understanding of veterinarians’ needs as they relate to AMR surveillance and management.

## 3. Results

### 3.1. Participant Demographics

**Figure 1:**
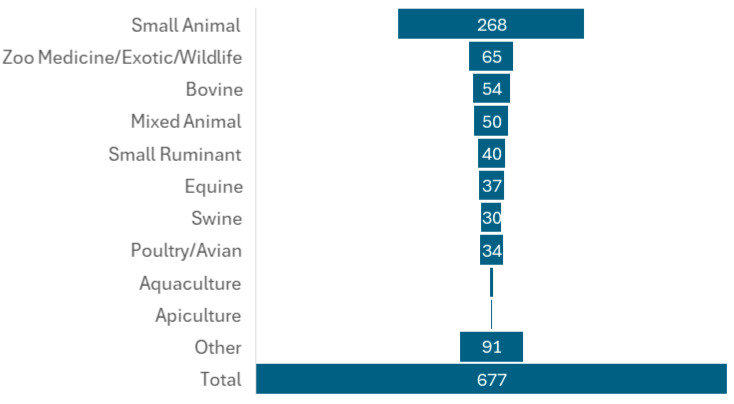
Distribution of survey participants by practice type.

The socio-demographic characteristics of the participants are summarized in Table 2 and Table 3. A total of 677 veterinarians from the United States completed the survey, which represents a 20% response rate of the survey sent out to 1916 participants. Even though the number of small animal vet respondents was slightly lower, post hoc power analysis resulted in 100% power to detect the expected 81% participation from the group. Other commodity group categories had higher participation than expected. Among them, 268 participants (39.59%) identified their practice type as “small animal” (Figure 1) Not all respondents answered every demographic question; however, of the 485 veterinarians who reported their age, 350 (73.17%) were between 30 and 59 years old, with ages ranging from 20 to over 70. In terms of gender, 477 respondents provided this information, with the majority being female (*n* = 294; 61.64%). Regarding professional experience, more than half of the 476 respondents (*n* = 249; 52.31%) reported having over 20 years of experience as veterinarians, while 40 respondents (8.40%) indicated they had less than five years of experience. Among the 601 respondents, 104 (17.29%) held a master’s degree, and 51 (8.49%) possessed a PhD. Of the 464 veterinarians, 121 (26.08%) had completed at least one residency program, and 201 (44.18%) had obtained at least one board certification. Furthermore, the majority of participants (*n* = 412; 88.03%) reported having pets, with 264 (30.84%) owning cats and 335 (39.14%) owning dogs.

### 3.2. Level of Comfort in Sharing the Types of AMR Data

Participants were invited to rank their comfort level regarding the sharing of geographic data, patient data, and antimicrobial susceptibility data related to AMR on a dashboard, ranging from extremely comfortable to extremely uncomfortable (see Table 4). More than 70% of respondents indicated that they were either extremely comfortable (*n* = 224; 40.89%) or somewhat comfortable (*n* = 98; 21.83%) with sharing state or zone-level data. Although this percentage decreased for sharing data at the county and congressional district levels, approximately 40% of participants still felt extremely comfortable sharing AMR data at these levels. Additionally, over 70% of veterinarians expressed that they were either extremely comfortable or somewhat comfortable sharing information about antimicrobial use and de-identified animal data. Furthermore, nearly 75% of respondents reported comfort in sharing antimicrobial susceptibility testing (AST) data, including details such as specimen types, AST profiles, and the types of AST panels utilized.

### 3.3. Perceived Level of Importance or Priority Regarding the AMR Issues

Veterinarians were surveyed to assess their views on the importance of sharing AMR data through a centralized dashboard (see Table 5). This initiative serves several significant purposes, including enhancing antimicrobial stewardship education, facilitating collaboration with the Clinical Laboratory Standards Institute (CLSI) for the development of veterinary antibiotic guidelines, conducting surveillance studies, and creating reporting tools. Additionally, the dashboard would aid in refining empirical treatment practices and monitoring antimicrobial usage in clinical settings. The results were compelling, with over 75% of respondents deeming all these activities as either extremely important or very important.

### 3.4. Level of Preference for Sharing Access to the AMR Dashboard with Various Institutions and Agencies

When asked to rank their preferences (ranked from highest preference, no preference, or lowest preference) for granting access to the dashboard for various institutions and agencies, over 70% of participating veterinarians expressed a strong preference for allowing access to veterinary colleges, teaching hospitals, veterinary diagnostic laboratories, and the USDA (see Table 6). Additionally, more than 60% of veterinarians surveyed indicated high preference ratings for sharing AMR dashboard access with the CDC (66.67%, *n* = 258), FDA (62.11%, *n* = 241), and the American Veterinary Medical Association (61.34%, *n* = 238). More than half of the respondents also showed a strong preference for granting access to WOAH (56.74%, *n* = 219) and CLSI (54.26%, *n* = 210). In contrast, the least preferred options were sharing access with the general public (21.50%, *n* = 83) and other potential audiences (15.18%, *n* = 58).

## 4. Discussion

This comprehensive cross-sectional survey involving 677 U.S. veterinarians revealed substantial support for establishing a centralized, veterinary medicine related, AMR dashboard, with strong endorsement for advanced tools to manage and mitigate AMR. The survey particularly captured veterinarians’ preferences for (a) types of dashboard data collection and dissemination, (b) objectives for overall use of the dashboard, and (c) potential regulation of governmental and stakeholder access.

The survey revealed a near-unanimous consensus on the importance of the proposed dashboard functionalities. Over 75% of respondents rated the following categories as either extremely or very important: (a) Antimicrobial Stewardship: Prioritizing education, guidance on off-label antimicrobial use, and collaboration with the Clinical and Laboratory Standards Institute (CLSI) for developing veterinary antibiotic guidelines; (b) Data and Surveillance: Supplying data for current and future surveillance studies, aiding in the development of reporting tools, and enabling the annual tracking of antimicrobial usage across different commodity groups; and (c) Clinical Practice: Assisting with empirical treatment practices or optimizing antibiotic use even before antimicrobial susceptibility testing (AST) results are available. This strong response indicates a clear, unmet need and a highly receptive user base. Such engagement is crucial for federal agencies aiming to sustain stakeholder participation and gather essential feedback to effectively address AMR surveillance strategies and dashboard development [12,32]. Moreover, the demand for educational components indicates that veterinarians want a dashboard that not only visualizes data but also converts data into practical guidance for better stewardship and professional development.

Concerns about data confidentiality, particularly when information is tied to specific farms, producers, or veterinarians, have been a major obstacle for implementing AMR dashboards [12,33]. This issue often limits data collection and sharing, undermining the effectiveness of surveillance systems. However, the survey results indicate that over 70% of veterinarians are comfortable sharing AMR data at the state or zone level and with de-identified animal information. This strong preference for aggregated, de-identified data indicates that a dashboard designed with these safeguards can overcome confidentiality issues, build trust, and encourage broader participation. This approach would allow for meaningful trend analysis without compromising individual privacy, creating a collaborative environment essential for comprehensive surveillance [33,34].

Veterinarians’ preferences for dashboard access clearly favor a collaborative, multi-institutional model. A significant majority of respondents support access for key academic (veterinary colleges, diagnostic laboratories), and federal (USDA, CDC, and FDA) stakeholders. This indicates a strong desire for a credible and authoritative system. Moreover, this collaborative perspective directly supports the “One Health” approach by the National Action Plan for Combating Antibiotic-Resistant Bacteria (CARB). The approach emphasizes integrated efforts across human, animal, and environmental health sectors, necessitating close collaboration among federal agencies like the CDC, FDA, USDA, and veterinary stakeholders [35]. This model should leverage academic and diagnostic institutions as central hubs for data input, analysis, and education, while federal agencies provide overarching guidance and integrate the data into national public health strategies. The AVMA can also be a crucial partner in facilitating adoption. To protect confidentiality, veterinarians preferred limited direct access to AMR dashboard data for the public and industry, instead providing data to these audiences through curated, aggregated reports. Having this multi-institutional framework, prioritizing trust and data quality, is essential for the dashboard’s impact and sustainability.

The overall survey received a greater number of respondents than expected. While the “small animal” practice group did not meet its calculated companion animal commodity sample size of 312, it did receive 268 responses, which represents approximately 85.9% of the expected size. This is considered a good representative population pool for the survey. In contrast, other commodity groups like mixed animal, equine, and food animal well exceeded their calculated sample sizes. The survey’s outreach methodology, while designed to maximize participation, introduced certain limitations that may impact the generalizability of the findings. The survey’s dissemination method, utilizing professional channels such as veterinary teaching hospitals, specialty organization listservs, and professional veterinary associations, may have introduced a sampling bias. Although this approach was effective in reaching a broad professional audience, it may have resulted in a self-selected group of veterinarians who are more engaged with or knowledgeable about AMR. Consequently, the findings may not be fully generalizable to the entire U.S. veterinary population. This potential for response bias and limited representativeness suggests that the level of support or specific preferences may be overestimated, underscoring the necessity for targeted future engagement. Although the survey garnered a substantial total of 677 respondents, the representation from certain specialized practice types was notably limited. For example, veterinarians primarily working in aquaculture and apiculture constituted only 0.89% (6 respondents) and 0.30% (2 respondents) of the total, respectively. This limited representation means that the specific preferences, challenges, and unique needs of these highly specialized sectors may not have been adequately captured. This underrepresentation could result in a dashboard design that overlooks or insufficiently addresses their distinct requirements, thereby compromising its comprehensive utility across all veterinary disciplines. Another key limitation of this study is the lack of detailed information regarding the geographical distribution of respondents. Without this crucial data, it is difficult to ascertain whether the survey’s findings are representative of all regions across the United States. Regional variations in antimicrobial use practices, pathogen prevalence, and regulatory environments could significantly influence veterinarians’ perspectives and priorities for an AMR dashboard. The absence of this geographical context limits the ability to assert the broader applicability of the findings across the diverse U.S. veterinary landscape. Therefore, while the current results offer strong initial guidance, future development phases must actively engage underrepresented groups and regions to ensure the dashboard’s utility and acceptance are truly nationwide and comprehensive by using a mixed-mode approach (online + offline) to enhance participation and improve representation. This underscores the critical importance of inclusive stakeholder engagement for public health initiatives.

In summary, this survey provides a strong foundation by outlining veterinarian preferences for a U.S. veterinary AMR dashboard. The survey results underscore the need for a collaborative, multi-institutional model, where a trusted and authoritative system—accessed by veterinary colleges, diagnostic laboratories, and federal agencies—translates surveillance data into practical guidance for antimicrobial stewardship and professional growth. To create a truly comprehensive and effective tool, future development must adopt an iterative, user-centered design approach. The next steps are to conduct qualitative research with diverse stakeholders, including farmers and animal owners, to capture a holistic understanding of their needs. This should be followed by a pilot phase with preferred institutional partners to rigorously test and refine a prototype. This strategic, multi-phase approach will ensure the dashboard is robust, trusted, and sustainable, ultimately maximizing its impact on animal and public health.

## Figures and Tables

**Table 1:** Sample size calculation.

Commodity Group	Number of Veterinarians in 2022	Proportion	Expected Response	Survey Sent to
Companion animal	62,400	0.81	312	1560
Mixed animal	3900	0.05	19.5	98
Equine	3700	0.05	18.5	93
Food animal	3600	0.05	18	90
Industry	3000	0.04	15	75
Total	76,600	1.00	383	1916

Population size (for finite population correction factor) (N): 76,600. Hypothesized % frequency of outcome factors in the population (*p*): 50% ± 5. Confidence limits as % of 100 (absolute ± %) (d): 5%. Design effect (for cluster surveys-DEFF): 1. Sample size (*n*) for various confidence levels. Confidence level (%) 95%. Sample size = 383.

**Table 2:** Socio-demographic information of the participants—veterinary practice type.

Practice Type	Number of Respondents (%)
Small Animal	268 (39.59)
Zoo Medicine/Exotic/Wildlife	65 (9.60)
Bovine	54 (7.98)
Mixed Animal	50 (7.39)
Small Ruminant	40 (5.91)
Equine	37 (5.47)
Swine	30 (4.43)
Poultry/Avian	34 (5.02)
Aquaculture	6 (0.89)
Apiculture	2 (0.30)
Other	91 (13.44)
Total	677 (100)

**Table 3:** Socio-demographic information of the participants—age, gender, years in practice, level of education, residencies, board certifications, pet ownership, and types of animals owned.

Category	Number (%)
**Age**	
20–29 years	19 (3.92)
30–39 years	113 (23.30)
40–49 years	123 (25.36)
50–59 years	114 (23.51)
60–69 years	90 (18.56)
70 or older	26 (5.36)
**Gender (number of respondents answered the question = 477)**	
Male	171 (35.85)
Female	294 (61.64)
Other	1 (0.21)
Prefer not to say	11 (2.31)
**Years in Practice**	
Less than 5	40 (8.40)
5 to 9	73 (15.34)
10 to 14	56 (11.76)
15 to 19	58 (12.18)
More than 20	249 (52.31)
**Education (number of respondents answered the question = 601)**	
MS	104 (17.30)
DVM/VMD or equivalent	446 (74.21)
PhD	51 (8.49)
**Residencies completed (number of respondents answered the question = 464)**	
Yes	121 (26.08)
No	343 (73.92)
**Board certifications (number of respondents answered the question = 464)**	
Yes	205 (44.18)
No	259 (55.82)
**Animal ownership (number of respondents answered the question = 468)**	
Yes	412 (88.03)
No	56 (11.97)
**Type of animal owned (number of responses = 856)**	
Cat	264 (30.84)
Dog	335 (39.14)
Horse	54 (6.31)
Goat	14 (1.64)
Sheep	7 (0.82)
Poultry	34 (3.97)
Cattle	19 (2.22)
Pig	5 (0.58)
Fish	40 (4.67)
Turtle	13 (1.52)
Other	71 (8.29)

**Table 4:** Number of responses and percentages of the level of comfort in sharing the types of AMR data in a dashboard.

AMR Data Type	Extremely Comfortable	Somewhat Comfortable	Neither Comfortable nor Uncomfortable	Somewhat Uncomfortable	Extremely Uncomfortable
**Geographic data**					
State region/zone-level AMR data	224 (49.89%)	98 (21.83%)	103 (22.94%)	13 (2.90%)	11 (2.45%)
County-level AMR data	196 (43.95%)	100 (22.42%)	106 (23.77%)	26 (5.83%)	18 (4.04%)
Congressional District-level AMR data	177 (39.95%)	87 (19.64%)	129 (29.12%)	29 (6.55%)	21 (4.74%)
**Patient data**					
Information related to antimicrobials administered after submitting samples for AST	202 (47.75%)	112 (26.48%)	83 (19.62%)	14 (3.31%)	12 (2.84%)
De-identified Host/animal information	200 (47.28%)	119 (28.13%)	77 (18.20%)	14 (3.31%)	13 (3.07%)
Information related to antimicrobials administered before submitting samples for AST	197 (46.68%)	118 (27.96%)	82 (19.43%)	14 (3.32%)	11 (2.61%)
Prior antimicrobial usage information in inpatient	184 (43.71%)	137 (32.54%)	77 (18.29%)	9 (2.14%)	14 (3.33%)
**AST data**					
Type of specimen submitted for AST	222 (54.01%)	90 (21.90%)	82 (19.95%)	6 (1.46%)	11 (2.68%)
Antimicrobial sensitivity patterns (e.g., bacterial species and drug data)	216 (52.55%)	103 (25.06%)	76 (18.49%)	5 (1.22%)	11 (2.68%)
Type of AST panel used	216 (52.05%)	95 (22.89%)	85 (20.48%)	8 (1.93%)	11 (2.65%)

**Table 5:** Number of responses and percentage of perceived level of importance or priority regarding the AMR issues.

AMR Issues	Extremely Important	Very Important	Moderately Important	Slightly Important	Not at All Important
Teaching and educating antimicrobial stewardship practices, including off-label use	259 (65.08%)	90 (22.61%)	38 (9.55%)	5 (1.26%)	6 (1.51%)
Sharing AMR data with the CLSI to develop guidelines for veterinary use	234 (58.79%)	101 (25.38%)	41 (10.30%)	17 (4.27%)	5 (1.26%)
Sharing AMR data for future and ongoing surveillance studies and reporting tool development	229 (57.25%)	112 (28.00%)	45 (11.25%)	10 (2.50%)	4 (1.00%)
Sharing AMR data for empirical treatment practices or prior to AST	202 (50.88%)	124 (31.23%)	53 (13.35%)	14 (3.53%)	4 (1.01%)
Sharing data relevant to tracking antimicrobial use in clinical practice, for each commodity group on an annual basis	190 (47.86%)	115 (28.97%)	68 (17.13%)	19 (4.79%)	5 (1.26%)

**Table 6:** Level of preference for sharing access to the veterinary AMR dashboard with various institutions and agencies.

Institutions	Highest Preference	No Preference	Lowest Preference
Colleges of Veterinary Medicine and Veterinary Teaching Hospitals	306 (78.87%)	78 (20.10%)	04 (1.03%)
Veterinary Diagnostic Laboratories	281 (72.61%)	93 (24.03%)	13 (3.36%)
United States Department of Agriculture (USDA)	278 (71.47%)	93 (23.91%)	18 (4.63%)
Centers for Disease Control (CDC)	258 (66.67%)	89 (23.00%)	40 (10.34%)
Food and Drug Administration (FDA)	241 (62.11%)	109 (28.09%)	38 (9.79%)
American Veterinary Medical Association (AVMA)	238 (61.34%)	137 (35.31%)	13 (3.35%)
World Organization for Animal Health (WOAH)	219 (56.74%)	128 (33.16%)	39 (10.10%)
Clinical and Laboratory Standards Institute (CLSI)	210 (54.26%)	155 (40.05%)	22 (5.68%)
Animal Agriculture Industry Groups	155 (40.05%)	182 (47.03%)	50 (12.92%)
General Public	83 (21.50%)	157 (40.67%)	146 (37.82%)
Other Potential Audiences/Stakeholders	58 (15.18%)	205 (53.66%)	119 (31.15%)

## Data Availability

The original contributions presented in this study are included in the article. Further inquiries can be directed to the corresponding author(s).

## Supplementary Materials

The following supporting information can be downloaded at: https://www.mdpi.com/article/10.3390/vetsci12100940/s1, File S1: Survey Instrument.

## Abbreviations

The following abbreviations are used in this manuscript:

AMR	Antimicrobial Resistance
APHIS	Animal and Plant Health Inspection Service
AST	Antimicrobial susceptibility testing
AVMA	American Veterinary Medical Association
AMU	Antimicrobial usage
ANIMUSE	ANImal antiMicrobial USE
CARB	Combating Antibiotic-Resistant Bacteria
CAVSNET	Companion Animal Veterinary Surveillance Network
CDC	Centers for Disease Control and Prevention
CLSI	Clinical and Laboratory Standards Institute
FDA	Food and Drug Administration
FSIS	Food Safety and Inspection Service
NDARO	National Database of Antibiotic Resistant Organisms
NAHLN	National Animal Health Laboratory Network
NARMS	National Antimicrobial Resistance Monitoring System
NIH	National Institutes of Health
NIAMRRE	The National Institute of Antimicrobial Resistance Research and Education
USDA	United States Department of Agriculture
Vet-LIRN	Veterinary Laboratory Investigation and Response Network
WGS	Whole genome sequencing
WHO	World Health Organization
WOAH	World Organization for Animal Health
