# Supplementary material for: Veterinarians’ Perspectives on the Antimicrobial Resistance (AMR) Dashboard: A Survey of Needs and Preferences to Inform Development
Source: Vet Sci. 2025 Sep 28;12(10):940. doi: 10.3390/vetsci12100940 (PMC12568220; doi:10.3390/vetsci12100940)
Supplement: Supplementary file 1 [file vetsci-12-00940-s001.zip › vetsci-3850561-supplementary.pdf]

AMR Dashboard Survey Instrument

Start of Block: Introduction and Instructions

Intro In response to Appropriations Bill 2021, the United States Department of Agriculture, Animal and Plant Health Inspection Service (USDA/APHIS) has provided cooperative agreement funding to multiple institutes to develop an antimicrobial resistance (AMR) dashboard tool to improve access to information on AMR in domesticated animals, including livestock, poultry, and companion animals.   This specific project, led by the University of Florida (UF), is one of the projects funded by the USDA-APHIS within a larger consortium to develop an AMR Dashboard Ecosystem for the use of monitoring trends in antimicrobial resistance patterns, detecting emerging resistance profiles, and better understanding relationships between antimicrobial use and animal health management practices and antimicrobial resistance. This UF project specifically aims to survey veterinarians to identify their concerns associated with large-scale curation of shared AMR surveillance data and, more importantly, to weigh in on the opinions of veterinarians by focusing on their needs, interests, and preferences for AMR data presentation on the dashboard tool.   The dashboard tool developed will provide data protection similar to the Confidential Information Protection and Statistical Efficiency Act. This survey is entirely anonymous, and all data will be stored in a secure digital environment. Only the cumulated results of the study will be used in publications, as needed. Although there are no direct benefits to the survey participants, a comprehensive AMR dashboard tool developed in collaboration with other institutes will greatly benefit the broader community, including the veterinarians. The University of Florida Institutional Review Board has designated this project as "exempt research" and authorized us to conduct this research (Protocol #ET00019861). This survey should not take more than 15 minutes to complete. If you have any questions or comments regarding this survey, please contact the Principal Investigator, Dr. Subhashinie Kariyawasam (skariyawasam@ufl.edu).

End of Block: Introduction and Instructions

Start of Block: Respondent Demographics

Q1 How would you categorize your practice (select all that apply)?

- [ ] Mixed Animal
- [ ] Bovine
- [ ] Small Ruminant
- [ ] Swine
- [ ] Poultry/Avian
- [ ] Small Animal
- [ ] Equine
- [ ] Zoo Medicine/Exotic/Wildlife
- [ ] Aquaculture
- [ ] Apiculture
- [ ] Other (please specify below)

____

Page Break

Q2 In what state is your residence/practice?

▼ Alabama ... I do not reside in the United States

Page Break

Q3 What is your age?

- 20-29
- 30-39
- 40-49
- 50-59
- 60-69
- 70 or older

Page Break

Q4 How many years have you been in practice?

- ◯ Less than 5
- ◯ 5 to 9
- ◯ 10 to 14
- ◯ 15 to 19
- ◯ More than 20

Page Break

Q5 What is your gender?

- ○ Female
- ○ Male
- ○ Prefer not to say
- ○ Other (please specify below)

____

Page Break

Q6 What level of education have you completed? (select all that apply)

- [ ] MS
- [ ] DVM/VMD or equivalent
- [ ] PhD

Page Break

Q7 Have you completed any additional residencies?

- ◯ No
- ◯ Yes (please specify below)

____

Page Break

Q8 Have you acquired any additional certifications (i.e., board certifications)?

- ○ No
- ○ Yes (please specify below) ____

Page Break

Q9 Do you personally own animals (i.e., animals intended for sport or companionship, and not food production)?

- No
- Yes

Page Break

Q10 Which animals do you own? (select all that apply)

- ☐ Cat
- ☐ Dog
- ☐ Horse
- ☐ Goat
- ☐ Sheep
- ☐ Poultry
- ☐ Cattle
- ☐ Pig
- ☐ Fish
- ☐ Turtle
- ☐ Other (please specify below)

____

End of Block: Respondent Demographics

Start of Block: Data Sharing

Q11 Describe your comfort level in sharing the following types of anti-microbial resistance (AMR) geographic data in a dashboard reporting tool:

	Extremely comfortable	Somewhat comfortable	Neither comfortable nor uncomfortable	Somewhat uncomfortable	Extremely uncomfortable
State region/zone-level AMR data	○	○	○	○	○
County-level AMR data	○	○	○	○	○
Congressional District-level AMR data	○	○	○	○	○

Page Break

Q12 Describe your comfort level in sharing the following types of anti-microbial resistance (AMR) patient data in a dashboard reporting tool:

	Extremely comfortable	Somewhat comfortable	Neither comfortable nor uncomfortable	Somewhat uncomfortable	Extremely uncomfortable
De-identified Host/animal information	○	○	○	○	○
Information related to antimicrobials administered before submitting samples for AST	○	○	○	○	○
Information related to antimicrobials administered after submitting samples for AST	○	○	○	○	○
Prior antimicrobial usage information in patient	○	○	○	○	○

Page Break

Q13 Describe your comfort level in sharing the following types of anti-microbial resistance (AMR) AST data in a dashboard reporting tool:

	Extremely comfortable	Somewhat comfortable	Neither comfortable nor uncomfortable	Somewhat uncomfortable	Extremely uncomfortable
Type of AST panel used	○	○	○	○	○
Type of specimen submitted for AST	○	○	○	○	○
Antimicrobial sensitivity patterns (e.g., bacterial species and drug data)	○	○	○	○	○

End of Block: Data Sharing

Start of Block: Data Importance/Priority

Q14 Describe your perceived level of importance/priority regarding the following AMR issues:

	Extremely important	Very important	Moderately important	Slightly important	Not at all important
Sharing AMR data for future and ongoing surveillance studies and reporting tool development	○	○	○	○	○
Sharing AMR data for empirical treatment practices or prior to AST	○	○	○	○	○
Sharing AMR data with the Clinical and Laboratory Standards Institute (CLSI) to develop guidelines for veterinary use	○	○	○	○	○
Sharing data relevant to tracking antimicrobial use in clinical practice, for each commodity group on an annual basis	○	○	○	○	○
Teaching and educating antimicrobial stewardship practices, including off-label use	○	○	○	○	○

End of Block: Data Importance/Priority

Start of Block: Access of Institutions/Agencies

Q15 Describe your level of preference for sharing access to the veterinary AMR dashboard with the following institutions and agencies:

	Highest preference	No preference	Lowest preference
Colleges of Veterinary Medicine and Veterinary Teaching Hospitals	◯	◯	◯
American Veterinary Medical Association (AVMA)	◯	◯	◯
United States Department of Agriculture (USDA)	◯	◯	◯
Food and Drug Administration (FDA)	◯	◯	◯
Centers for Disease Control (CDC)	◯	◯	◯
World Organization for Animal Health (WOAH) (formerly OIE)	◯	◯	◯
Clinical and Laboratory Standards Institute (CLSI)	◯	◯	◯
Veterinary Diagnostic Laboratories	◯	◯	◯
Animal Agriculture Industry Groups	◯	◯	◯
General Public	◯	◯	◯
Other Potential Audiences/Stakeholders	◯	◯	◯

End of Block: Access of Institutions/Agencies
